# LIS1 Regulates Osteoclast Formation and Function through Its Interactions with Dynein/Dynactin and Plekhm1

**DOI:** 10.1371/journal.pone.0027285

**Published:** 2011-11-03

**Authors:** Shiqiao Ye, Tristan W. Fowler, Nathan J. Pavlos, Pei Ying Ng, Kai Liang, Yunfeng Feng, Minghao Zheng, Richard Kurten, Stavros C. Manolagas, Haibo Zhao

**Affiliations:** 1 Center for Osteoporosis and Metabolic Bone Diseases, Division of Endocrinology and Metabolism, Department of Internal Medicine, University of Arkansas for Medical Sciences and the Central Arkansas Veterans Healthcare System, Little Rock, Arkansas, United States of America; 2 Department of Physiology and Biophysics, University of Arkansas for Medical Sciences, Little Rock, Arkansas, United States of America; 3 Center for Orthopedic Research, School of Surgery, University of Western Australia, Perth, Australia; 4 Department of Dermatology, University of Arkansas for Medical Sciences, Little Rock, Arkansas, United States of America; 5 Department of Internal Medicine, Washington University School of Medicine, St. Louis, Missouri, United States of America; University of Muenster, Germany

## Abstract

Microtubule organization and lysosomal secretion are both critical for the activation and function of osteoclasts, highly specialized polykaryons that are responsible for bone resorption and skeletal homeostasis. Here, we have identified a novel interaction between microtubule regulator LIS1 and Plekhm1, a lysosome-associated protein implicated in osteoclast secretion. Decreasing LIS1 expression by shRNA dramatically attenuated osteoclast formation and function, as shown by a decreased number of mature osteoclasts differentiated from bone marrow macrophages, diminished resorption pits formation, and reduced level of CTx-I, a bone resorption marker. The ablated osteoclast formation in LIS1-depleted macrophages was associated with a significant decrease in macrophage proliferation, osteoclast survival and differentiation, which were caused by reduced activation of ERK and AKT by M-CSF, prolonged RANKL-induced JNK activation and declined expression of NFAT-c1, a master transcription factor of osteoclast differentiation. Consistent with its critical role in microtubule organization and dynein function in other cell types, we found that LIS1 binds to and colocalizes with dynein in osteoclasts. Loss of LIS1 led to disorganized microtubules and aberrant dynein function. More importantly, the depletion of LIS1 in osteoclasts inhibited the secretion of Cathepsin K, a crucial lysosomal hydrolase for bone degradation, and reduced the motility of osteoclast precursors. These results indicate that LIS1 is a previously unrecognized regulator of osteoclast formation, microtubule organization, and lysosomal secretion by virtue of its ability to modulate dynein function and Plekhm1.

## Introduction

Osteoclasts are terminally differentiated polykaryons that are uniquely capable of digesting calcified bone matrix. They are formed by fusion of mononuclear precursors of the monocyte/macrophage lineage [1], [2]. Receptor activator of nuclear factor kappa B (NF-κB) ligand (RANKL) and macrophage colony-stimulating factor (M-CSF) are the essential cytokines for osteoclastogenesis[3]; and NFATc1 is the master transcription factor responsible for osteoclast differentiation and function. NFATc1 is induced by RANKL and co-activated by immunoglobulin-like receptors and their associated adapter proteins [4], [5]. As they mature, osteoclasts undergo dramatic reorganization of their cytoskeleton. Filamentous actin (F-actin) is first organized into podosomes, highly dynamic structures that mediate cell adhesion and migration of osteoclasts. When osteoclasts are cultured on glass or plastic, individual podosomes are clustered and expand to the cell periphery to form a stable “podosome belt” [6], [7]. When osteoclasts are cultured on bone, F-actin forms a ring-like structure (actin-ring) at the sealing zone, a tight adhesion structure where the osteoclast plasma membrane is juxtaposed to bone [8]. The sealing zone surrounds a specialized plasma membrane domain, the ruffled border, thus forming an isolated resorptive microenvironment between the osteoclast and the underlying bone matrix. The ruffled border is generated by the fusion of secretory vesicles with the bone-apposing plasma membrane. During this process, protons and lysosomal enzymes (predominantly cathepsin K) are vectorially secreted into the resorption lacuna to dissolve bone mineral and digest organic matrix, respectively [9]. It has been recently shown that the network of microtubules regulates podosome patterning in osteoclasts and thus is essential for osteoclast spreading and the sealing zone formation [10], [11]. One of the mechanisms of microtubule stabilization in osteoclasts is regulated by tubulin acetylation. This process is controlled by a Rho-mDia2-HDAC6 pathway where activation of small GTPase Rho promotes the deacetylation of tubulin through the activation of Rho downstream effector mDia2 and the histone deacetylase HDAC6. The protein tyrosine kinase Pyk2 and Cbl family members of adaptor proteins regulate actin-ring formation and bone resorption, at least in part, through their modulation of Rho and HDAC6 activities [12], [13].

During the last few years, genetic studies of patients with osteopetrosis as well as naturally occurring mutation or gene-targeting in mice have elucidated important regulatory proteins that control osteoclastic bone resorption [3], [14]. Among these proteins, carbonic anhydrase II, a3 subunit of vacuolar proton pump, Clc-7 chloride channel, OSTM1 (β subunit of Clc-7) are essential for handling proton generation and acidification of the resorption lacuna. Cathepsin K is crucial for bone degradation. More recently, mutations of the PLEKHM1 gene have been identified as the cause of the osteopetrotic *ia/ia* (incisors absent) rat as well as a subset of patients with intermediate osteopetrosis [15]. The *ia/ia* rat exhibits generalized osteopetrosis and delay in tooth eruption that is inherited in an autosomal recessive manner [16]. Osteoclasts in *ia/ia* rats exhibit abnormal sealing zone formation and an intrinsic defect in ruffled border formation with accumulation of intracellular lysosomal enzymes [17], reflecting dysfunctions in intracellular vesicular trafficking pathways. Recent segregation analysis has localized the gene responsible for these abnormalities to rat chromosome 10q32.1 [18]. Sequence analysis of rat and human genomes has uncovered mutations in the PLEKHM1 gene in *ia/ia* rats and in a family with an intermediate form of osteopetrosis. Consistently, osteoclasts derived from peripheral blood monocytes from these individuals have impaired resorptive activity [15]. Plekhm1^R714C^ heterozygous mutation impairs vesicular acidification and increases TRAP secretion in osteoclasts [19]. Overexpression studies in HEK293 and osteoclast-like cells derived from the Raw 264.7 macrophages demonstrated that Plekhm1 is co-localized with Rab7 at late endosomes and lysosomes. Furthermore, a dominant negative mutant of Rab7 disrupts the association of Plekhm1 with lysosomes. Taken together, these findings suggest that Plekhm1 is a component of Rab7-regulated late endosomal trafficking in osteoclasts [15] and possibly in other cells as well [20]. However, the mechanism(s) by which Plekhm1 regulates this process remains unknown.

Haploinsufficiency of LIS1 (official symbol PAFAH1b1, for platelet-activating factor (PAF) acetylhydrolase isoform 1b subunit 1) causes lissencephaly, a severe human developmental brain disorder manifested by a smooth cerebral surface and disorganized cortical layers due to incomplete neuronal migration [21], [22]. LIS1-null mice are embryonic lethal at the implantation stage and heterozygous mice exhibit delayed neuronal migration and defects in cortical development [23]. Biochemical and genetic studies have revealed that LIS1, together with its binding proteins NDE1/NDEL1, form an evolutionary conserved pathway that regulates microtubule dynamics and the function of the retrograde molecular motor, cytoplasmic dynein. Indeed LIS1 and NDE1/NDEL1 are required in several dynein-mediated processes including nuclear and centrosome positioning and movement in migrating neuronal and non-neuronal cells; chromosome alignment and mitotic spindle orientation in proliferating cells; intracellular vesicular transport; and neuronal growth cone advance [24], [25]. LIS1 also serves as the non-catalytic β subunit of PAFAH 1b in complex with homo- or hetero-dimers of the catalytic subunits α1 and α2 [22], [26]. LIS1 may regulate the activity or the localization of PAFAH 1b complex which inactivates intracellular PAF by removing the acetyl group at the sn-2 position of the glycerol backbone of PAF [27]. A previous report has implicated PAF as an important autocrine factor for osteoclast survival and function. PAF receptor deficient mice are protected from osteoporosis following ovariectomy [28]. Given the important role of both microtubules and PAF in osteoclast biology we hypothesized that LIS1 may regulate bone homeostasis. The skeletal phenotypes of lissencephaly patients and the LIS1 heterozygous knockout mice have not been defined so far.

In the present report we have identified that LIS1 interacts with Plekhm1. LIS1 also forms complexes with dynein/dynactin in osteoclasts. Decreasing LIS1 expression by shRNA results in an aberrant M-CSF and RANKL signaling; altered microtubule organization; the perinuclear accumulation of lysosomes around the nucleus; and a decrease in osteoclast precursor proliferation, survival, motility and cathepsin K secretion. Thus, loss of LIS1 expression dramatically attenuates the formation of multinucleated osteoclasts and the bone resorption function of the mature cells by interfering with microtubule/dynein and Plekhm1pathways.

## Results

### LIS1 and Plekhm1 interact in osteoclasts

**Figure 1:**
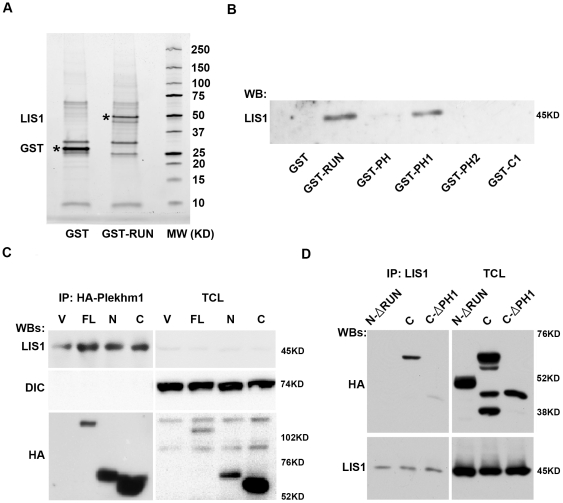
Plekhm1 is a novel LIS1-interacting protein in osteoclasts. (A and B) Endogenous LIS1 binds to RUN and PH1 domains of Plekhm1. GST and GST fusion proteins of individual domain of mouse Plekhm1 were expressed in *E. coli* and purified. Proteins bound to glutathione-Sepharose beads were incubated with mature osteoclast lysate, and co-purified proteins were visualized by Coomassie blue staining and identified by mass spectrometry (A) or immunoblotting (B). (C) HA tagged Plekhm1 is co-immunoprecipitated with endogenous LIS1 from mature osteoclast lysate. Bone marrow macrophages were retrovirally transduced with empty vector (V), full-length (FL), N-terminal half (N), and C-terminal half (C) of mouse Plekhm1. Mature osteoclasts were lysed and subjected to immunoprecipitation experiments with anti-HA antibody. Immunoprecipitated proteins were then analyzed by western blotting with anti-LIS1, anti-dynein intermediate chain (DIC) and anti-HA antibodies, respectively. (D) RUN and PH1 domains of Plekhm1 mediate its interaction with LIS1. Bone marrow macrophages were retrovirally transduced with N-terminal half of Plekhm1 without RUN domain (N-ΔRUN), C-terminal half (C) and C-terminal half depleted in PH1 domain (C-ΔPH1). Endogenous LIS1 was Immunoprecipitated by a rabbit polyclonal antibody from mature osteoclasts and the binding of Plekhm1 was detected by monoclonal anti-HA antibody. The multiple low molecular-weight bands shown in the TCL HA blot for C-terminal Plekhm1 are probably due to the degradation of the protein. MW, molecular mass; WB, Western blot; IP, immunoprecipitation; TCL, total cell lysate.

Plekhm1 is a cytoplasmic protein containing a RUN (RPIP8/UNC-14/NESCA) domain, two PH (Pleckstrin Homology) domains and a lipid binding and responsive C1 (protein kinase C conserved region 1) domain (Figure S1). We (Figure S2) and others [15] have previously demonstrated that Plekhm1 is associated with lysosomes in osteoclasts and regulates cathepsin K secretion. However, the precise functions of Plekhm1 remain unknown. To better understand how Plekhm1 might regulate osteoclast activity we first sought to identify its potential binding proteins. The GST fusion proteins of individual domains of mouse Plekhm1 were used to pull-down associated proteins from mature murine osteoclast lysates. As shown in Figure 1A, a ∼50 kDa band appeared specifically in GST-RUN domain pull-down but not in that of GST, as visualized by Coomassie blue staining. Mass spectrometry analysis of proteins in this band revealed that it contained both Plekhm1 RUN domain and LIS1. When the pull-downs from each Plekhm1 domain were probed by western blot with anti-LIS1 antibody, LIS1 was associated with both the RUN and PH1 domains of Plekhm1 (Figure 1B). The interaction between LIS1 and Plekhm1 was further confirmed by co-immunoprecipitation of endogenous LIS1 with HA tagged full length Plekhm1, the N-terminal half of Plekhm1 which contains the RUN domain and the C-terminal half of Plekhm1 which contains the PH1, PH2 and C1 domains (Figure 1C, upper panels). In contrast, endogenous dynein intermediate chain (DIC) was not immunoprecipitated with HA-Plekhm1, indicating that DIC did not interact with Plekhm1 directly (Figure 1C, middle panels). To further map which part(s) of Plekhm1 interact with LIS1, we retrovirally transduced a series of truncated fragments of murine Plekhm1 into bone marrow macrophages (BMMs) which were further cultured with M-CSF and RANKL for 5 days to generate osteoclasts (Figure 1D). While immunoprecipitated endogenous LIS1 was associated with the C-terminal half of Plekhm1, deletion of the RUN and/or PH1 domains abolished Plekhm1-LIS1 interaction (Figure 1D). Thus, the data from these GST pull-down and reciprocal immunoprecipitation experiments demonstrate that Plekhm1 binds to LIS1 through both RUN and PH1 domains.

### LIS1 is indispensible for osteoclast formation and function

**Figure 2:**
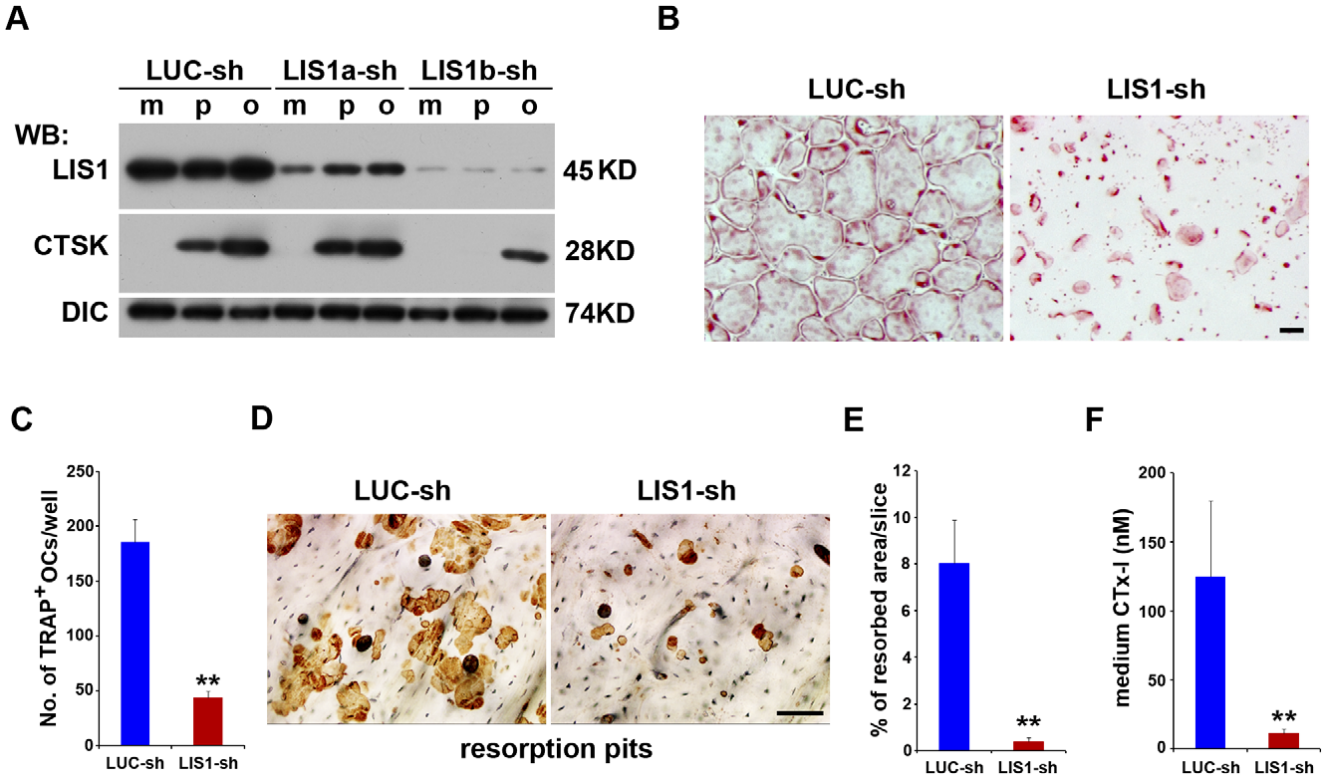
LIS1 is essential for osteoclast formation and function. (A) Knockdown of LIS1 expression by lentivirus mediated shRNAs. Bone marrow macrophages were transduced with lentiviral vectors expressing a control shRNA (LUC-sh) or LIS1 specific shRNAs (LIS1a-sh and LIS1b-sh), respectively. Bone marrow macrophages (m), pre-osteoclasts (p) and mature osteoclasts (o) were lysed and analyzed by western blotting. (B) LIS1 down-regulation attenuates multinucleated TRAP^+^ osteoclast formation. Scale bar  = 10 µm. (C) The number of TRAP^+^, multinucleated osteoclasts per well of 48-well plate were counted, n = 6. (D) Decreased LIS1 expression blocks osteoclastic bone resorption. Osteoclasts were cultured on cortical bovine bone slices and resorption pits were labeled with peroxidase-conjugated wheat germ agglutinin. Scale bar  = 10 µm. (E) The percentage of resorbed area/bone slice as measured by Ossteomeasure software. n = 6. (F) CTx-I level in culture medium was measured by ELISA (E), n = 6. WB, Western blot. = . ** p<0.01 vs LUC-sh by Student's t-test.

To elucidate the role of LIS1 in osteoclasts we next proceeded to knock down its expression by lenti-virus mediated shRNA expression. The knock-down efficiency was validated by western blotting using a monoclonal antibody specific to LIS1 (Figure 2A, upper panel). Western blots for cathepsin K and DIC served as a marker of osteoclast differentiation and loading control, respectively (Figure 2A, middle and lower panels). LIS1 protein levels were potently suppressed in the LIS1b shRNA (LIS1b-sh) transduced BMMs, pre-osteoclasts and mature osteoclasts as compared with those in control cells transduced with shRNA targeting fire fly luciferase (LUC-sh). In contrast, LIS1a shRNA (LIS1a-sh) expression resulted in only a modest reduction of LIS1 protein level. The osteoclast formation and cathepsin K expression in LIS1a-sh expressing cells were indistinguishable from those of control LUC-sh cells (Figure 2A and data not shown). We thereafter used LIS1b-sh, designated now as LIS1-sh, to inhibit LIS1 expression in osteoclasts in the following experiments. Decreased LIS1 expression by LIS1-sh dramatically attenuated the formation of multinucleated osteoclasts as revealed by tartrate-resistant acid phosphatase (TRAP) staining and quantification (Figure 2B and 2C), and a significant decrease in cathepsin K expression in LIS1b-sh expressing osteoclasts (Figure 2A, middle panel). When cultured on bone slices, the total area of resorption pits was significantly reduced in LIS1-sh expressing osteoclasts as compared with control cells (Figure 2D and 2E). More importantly, the concentration of CTx-I, fragment of collagen released from bone into the medium, was 10 times lower in LIS1-sh knockdown osteoclasts (Figure 2F). These effects were not due to an off-target phenomenon caused by overexpression of shRNAs, because expression of an independent LIS1 shRNA, which targets to a different site of LIS1 mRNA (see Materials and Methods), reproduced a similar result (Figure S3). Thus, LIS1 is essential for both osteoclast formation and function.

### LIS1 controls proliferation, survival and differentiation of osteoclast precursors via modulating M-CSF and RANKL signaling pathways and NFATc1 expression

**Figure 3:**
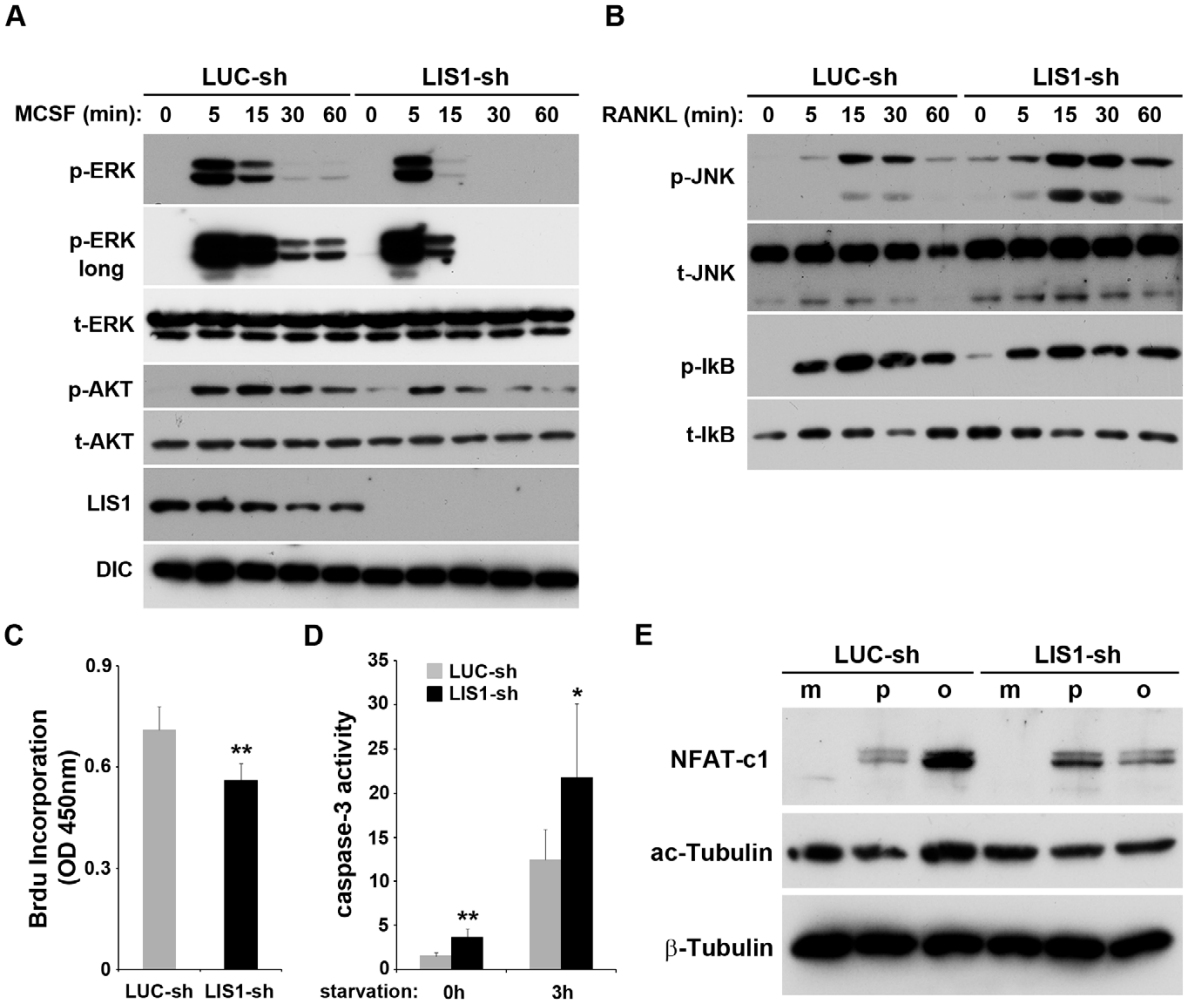
LIS1 controls macrophage proliferation, differentiation and survival via modulating M-CSF and RANKL signaling pathways and NFATc1. (A) Low level of LIS1 inhibits M-CSF induced ERK and AKT activation. LUC-sh and LIS1-sh transduced BMMs were serum and cytokine starved for 12 hours before stimulated with 50 ng/ml M-CSF for indicated time. ERK and AKT activation were detected by western blots with anti-phospho-ERK (p-ERK) and anti-phospho-AKT (p-AKT) antibodies. The knockdown efficiency was examined by LIS1 western blot. Western blots for total ERK (t-ERK), AKT (t-AKT) and DIC serve as loading controls. (B) Depletion of LIS1 results in sustained RANKL induced JNK activation without changing in NF-κB activation as determined by western blots with anti-phospho-JNK (p-JNK) and anti-phospho-IκB (p- IκB) antibodies. Total JNK (t-JNK) and IκB (t- IκB) serve as controls. (C) LIS1 knockdown BMMs are less proliferative, as measured by Brdu incorporation ELISA kit. (D) Loss of LIS1 accelerates osteoclast apoptosis. Pre-osteoclasts were either untreated or were serum and cytokine starved for 3 hours. Apoptosis was assessed by a fluorometric caspase-3 activity assay. (E) LIS1-depletiojn leads to decreased NFATc1 expression in osteoclasts. Bone marrow macrophages (m), pre-osteoclasts (p), and mature osteoclasts (o) were lysed and the level of NFATc1 and acetylated tubulin (ac-tubulin) was detected by Western blots. β-tubulin serves as a loading control. * p<0.05, ** p<0.01 vs LUC-sh by Student's t-test.

The compromised osteoclast formation in LIS1 knockdown BMMs could be the result of aberrant proliferation, differentiation, and/or apoptosis of osteoclast precursors and mature cells. To identify the mechanisms by which LIS1 regulates osteoclastogenesis we therefore determined whether LIS1 modulates the downstream signaling pathways of M-CSF and RANKL, the two critical cytokines regulating these processes. As shown in Figure 3A, M-CSF induced prolonged ERK and AKT activation in control BMMs, which has been shown to be required for macrophage proliferation and osteoclast survival [29], [30]. These effects were significantly reduced in LIS1 knockdown BMMs. Silencing of LIS1 also resulted in a sustained JNK activation in response to RANKL, which has been shown recently to induce osteoclast apoptosis [31]. On the other hand, RANKL-stimulated NFκB activation, as demonstrated by the phosphorylation of IκB was not affected by the LIS1-depletion (Figure 3B). Consistent with an important role of ERK and AKT pathways in macrophages, LIS1-deficient BMMs exhibited a decreased proliferative capacity as measured by BrdU incorporation rate (Figure 3C). In line with the aberrant JNK activation, LIS1 down-regulation correlated with an increase in pre-osteoclast apoptosis under both basal and starvation-induced conditions, as monitored by caspase-3 activity (Figure 3D) and nuclear staining (Figure S4). Since NFATc1 is an essential transcription factor for osteoclast differentiation we went on to test whether LIS1 regulates NFATc1 expression. As shown in Figure 3E, NFATc1 levels were markedly decreased in LIS1 knockdown mature osteoclasts whereas tubulin acetylation, which has been shown to modulate microtubules organization and mature osteoclast spreading, was only slightly reduced as compared with control cells. Collectively, these data indicate that LIS1 controls proliferation, survival and differentiation of osteoclast precursors by regulating M-CSF and RANKL signaling pathways and NFATc1.

### LIS1 is essential for the motility of osteoclast precursors and cathepsin K secretion

**Figure 4:**
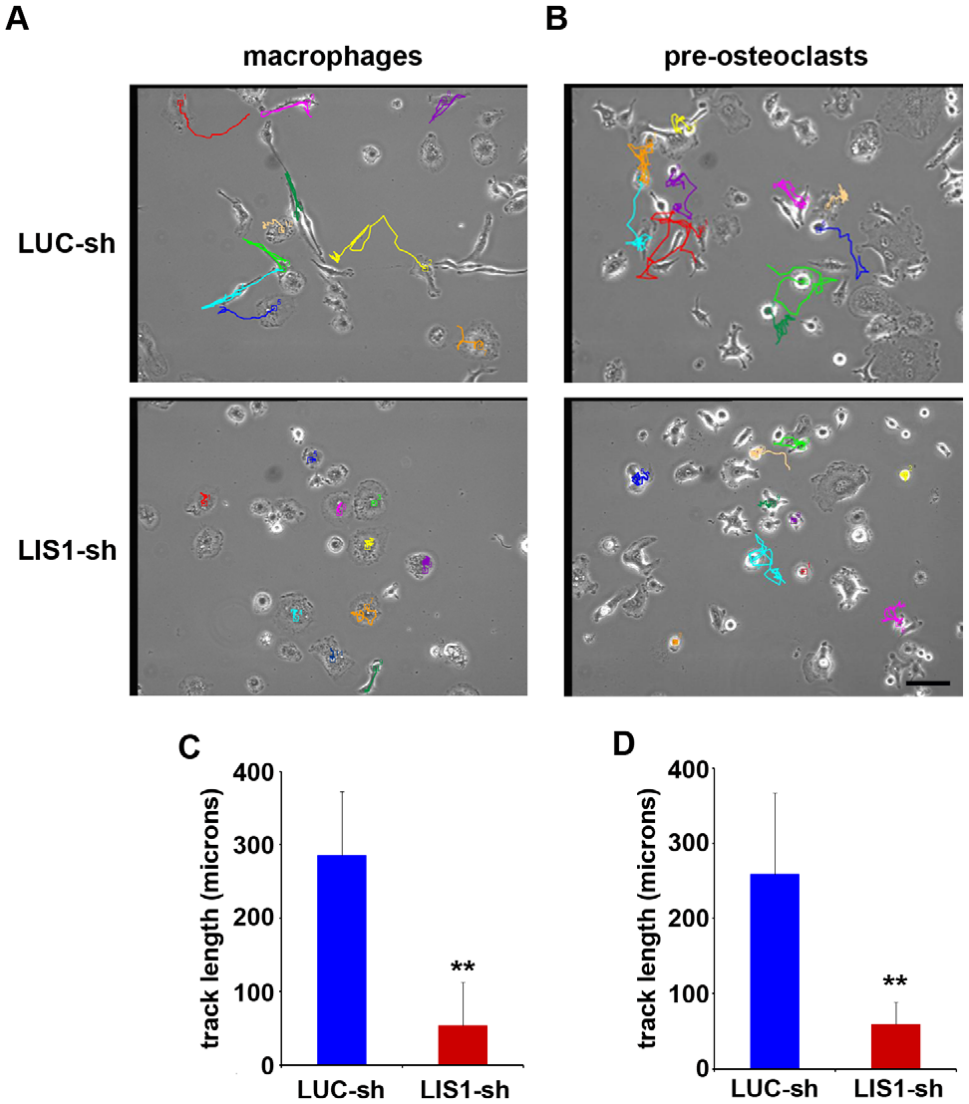
LIS1 regulates macrophage and pre-osteoclast motility. BMMs (A) and pre-osteoclasts (B) were cultured in Bioptechs dishes and the cells were imaged by time-lapse video microscopy. Images were obtained using Scion imaging software at 10 minute intervals for 8 hours. The 8-hour movements of ten motile osteoclast precursors in each cell group were tracked and analyzed with ImageJ MTrackJ plug-in (A and B). (C and D) Cumulative length of each track was depicted as microns displaced over 48 images. ** p<0.01 vs LUC-sh by Student's t-test. Scale bar  = 100 µm.

LIS1 has been shown to modulate cytoplasmic dynein function in several cellular processes such as neuronal migration, mitosis and intracellular membrane trafficking, including lysosome distribution [32]–[34]. We next investigated whether LIS1 is involved in pre-osteoclast migration by using time-lapse video microscopy on living cells. The 8-hour movements of ten motile macrophages and pre-osteoclasts in LUC-sh and LIS1-sh expressing cells were tracked and analyzed with ImageJ MTrackJ plug-in (National Institutes of Health) (Figure 4A and 4B). Quantitative analysis of cumulative track lengths revealed a striking 5-fold decrease in the cell motility of LIS1-depleted macrophages and pre-osteoclasts as compared to control cells (Figure 4C and 4D).

**Figure 5:**
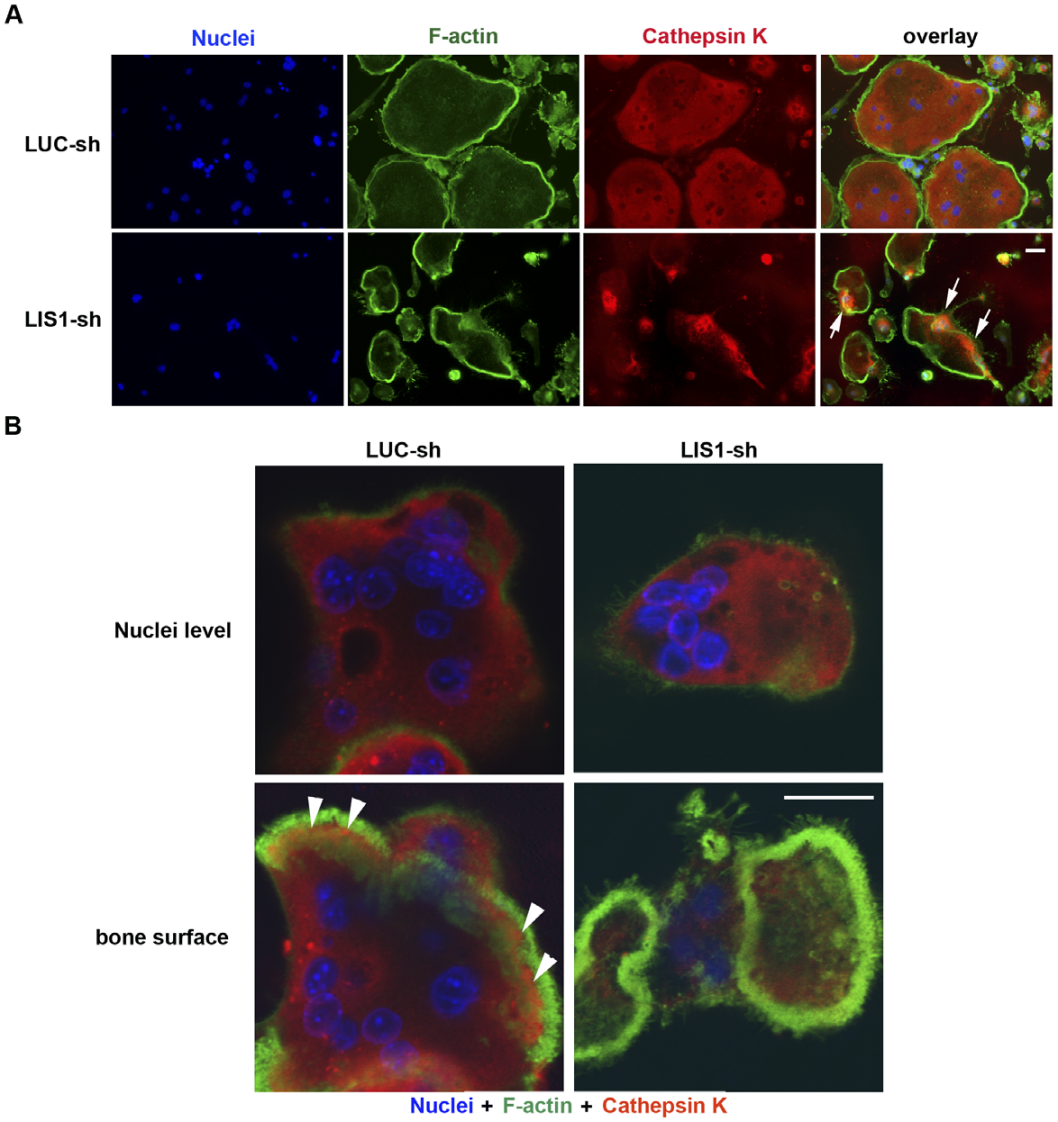
LIS1 is essential for cathepsin K secretion in osteoclasts. (A and B) Decreased LIS1 expression inhibits cathepsin K secretion in osteoclasts. F-actin and Cathepsin K in osteoclasts cultured on glass coverslips (A) and cortical bovine bone slices (B) were labeled by phalloidin and monoclonal anti-Cathepsin K antibody, respectively. Arrows in (A) demonstrate the aggregation of cathepsin K around the nucleus in LIS-1 knockdown osteoclasts. Arrow heads in (B) indicate the secretion of cathepsin K into the resorption lacuna inside the actin-rings in control osteoclasts. Scale bars  = 10 µm.

To determine whether LIS1 regulates osteoclast lysosomal trafficking and cathepsin K secretion, two processes that are essential for osteoclastic bone resorption, LUC-sh and LIS1-sh transduced BMMs were cultured on glass coverslips or cortical bovine bone slices with M-CSF and RANKL for 5 days. Mature osteoclasts were fixed and processed for the labeling with anti-cathepsin K antibody by immunofluorescence. In contrast to a dispersed distribution pattern of lysosomes in control osteoclasts (Figure 5A upper panels), LIS1 knockdown increased the density of cathepsin K positive lysosomes around the nucleus (Figure 5A lower panels). The secretion of the enzyme into the resorption lacuna in resorbing osteoclasts cultured on bone was greatly reduced as compared to control culture (Figure 5B lower panels). Taken together, these findings indicate that LIS1 is critical for osteoclast function through regulating its motility and cathepsin K secretion.

### LIS1 regulates osteoclast microtubule organization through dynein/dynactin motor complex

**Figure 6:**
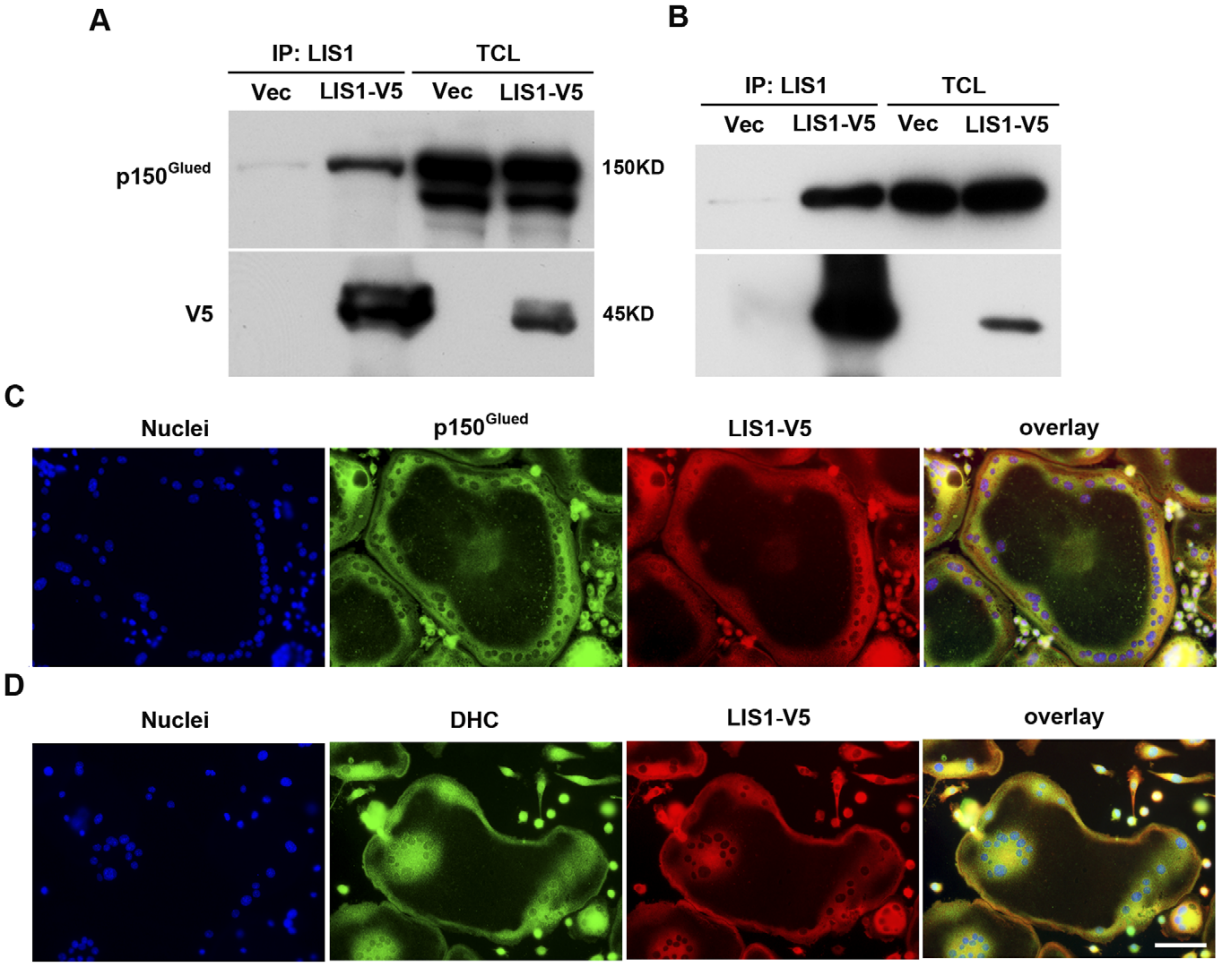
LIS1 interacts with dynein/dynactin complex in osteoclasts. (A and C) V5 tagged LIS1 (LIS1-V5) binds to and co-localizes with endogenous p150^glued^ subunit of dynactin complex in mature osteoclasts. Bone marrow macrophages were retrovirally transduced with empty vector (Vec) and LIS1-V5. Mature osteoclasts were lysed and subjected to immunoprecipitation experiments with anti-LIS1 antibody. Immunoprecipitated proteins were analyzed by Western blotting with anti- p150^glued^ and anti-V5 antibodies, respectively (A). Osteoclasts cultured on glass coverslips were fixed and processed for immunofluorescence with anti-V5 and p150 antibodies (C). (B and D) LIS1 binds to DIC and co-localizes with dynein heavy chain (DHC) in mature osteoclasts. WB, Western blot; IP, immunoprecipitation; TCL, total cell lysate. Scale bar  = 10 µm.

LIS1 has been previously shown to regulate microtubule dynamics and intracellular transportation through its association with the cytoplasmic dynein/dynactin motor complex in neuronal cells [33], [35]. To uncover the mechanisms by which LIS1 controls osteoclast function we next examined whether LIS1 is involved in the regulation of dynein/dynactin in osteoclasts. To this end, we over-expressed V5 tagged mouse LIS1 (LIS1-V5) by retroviral transduction in BMMs. BMMs were then cultured with M-CSF and RANKL for 5 days to generate mature osteoclasts. The cells were lysed and immunoprecipitated with anti-LIS1 polyclonal antibody. Endogenous p150^glued^ subunit of dynactin complex and DIC were co-precipitated with LIS1-V5. Empty vector transduced cells served as negative control (Figure 6A and 6B). Furthermore, p150^glued^ and dynein heavy chain (DHC) were co-localized with LIS1-V5 in the cytoplasm around the nucleus and peripheral podosome rings in osteoclasts cultured on glass coverslips (Figure 6C, 6D, and Figure S5), indicating that LIS1 is a component of the dynein/dynactin pathway in osteoclasts.

**Figure 7:**
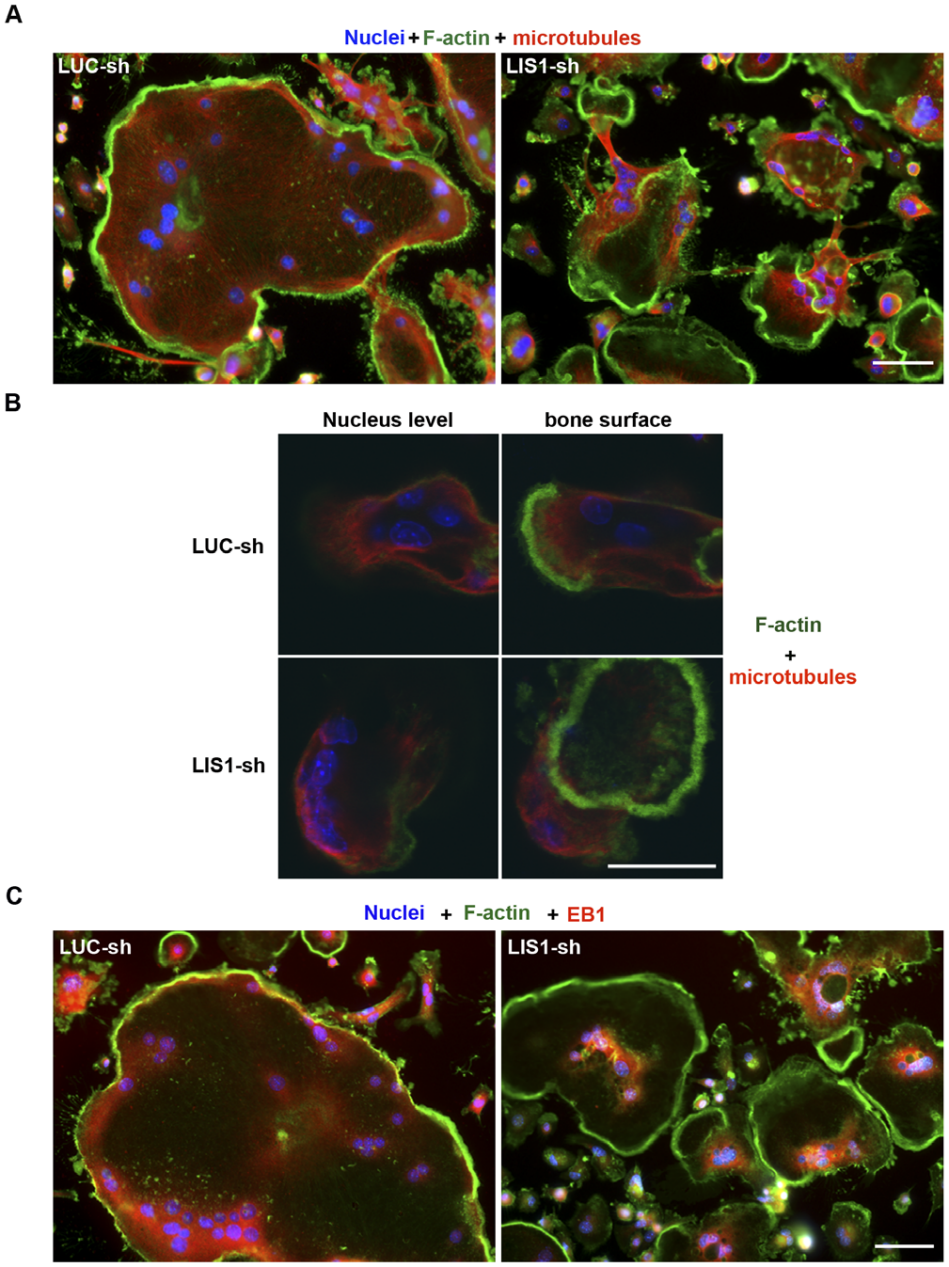
LIS1 regulates microtubule organization and EB1 distribution in osteoclasts. (A and B) LIS1 reduction induces condensed microtubules focused around nucleus. Filamentous actin (F-actin) and microtubules in osteoclasts cultured on glass coverslips (A) and cortical bovine bone slices (B) were labeled by Alexa-488 conjugated phalloidin and anti-tubulin antibody and were examined by conventional (A) and laser confocal microscopy (B), respectively. (C) EB1 is clustered around the nucleus and is less transported by dynein to the cell periphery in LIS1 knockdown osteoclasts (right panel) than in control cells (left panel). Cells were labeled by Hoechst 33258, phalloidin and anti-EB1 monoclonal antibody, respectively. Scale bars  = 10 µm.

Given that microtubule organization/stabilization is important for osteoclast activation and function, we then examined the microtubule distribution in LIS1-silenced osteoclasts. In control osteoclasts, microtubules were evenly distributed, radiating from the cytoplasm region around the nucleus to the peripheral podosome belts in osteoclasts cultured on glass (Figure 7A, left panel). In osteoclasts resorbing bone, microtubules were localized at the cortex of basal plasma membrane and over the ruffled membrane inside the actin-rings (Figure 7B, upper panels). In contrast, microtubules in osteoclasts lacking LIS1 were morphologically disorganized, often clustering around the nucleus and failing to extend to the podosome belts in spreading osteoclasts on glass (Figure 7A, right panel) and the ruffled membrane in bone resorbing osteoclasts on bone (Figure 7B, lower panels).

**Figure 8:**
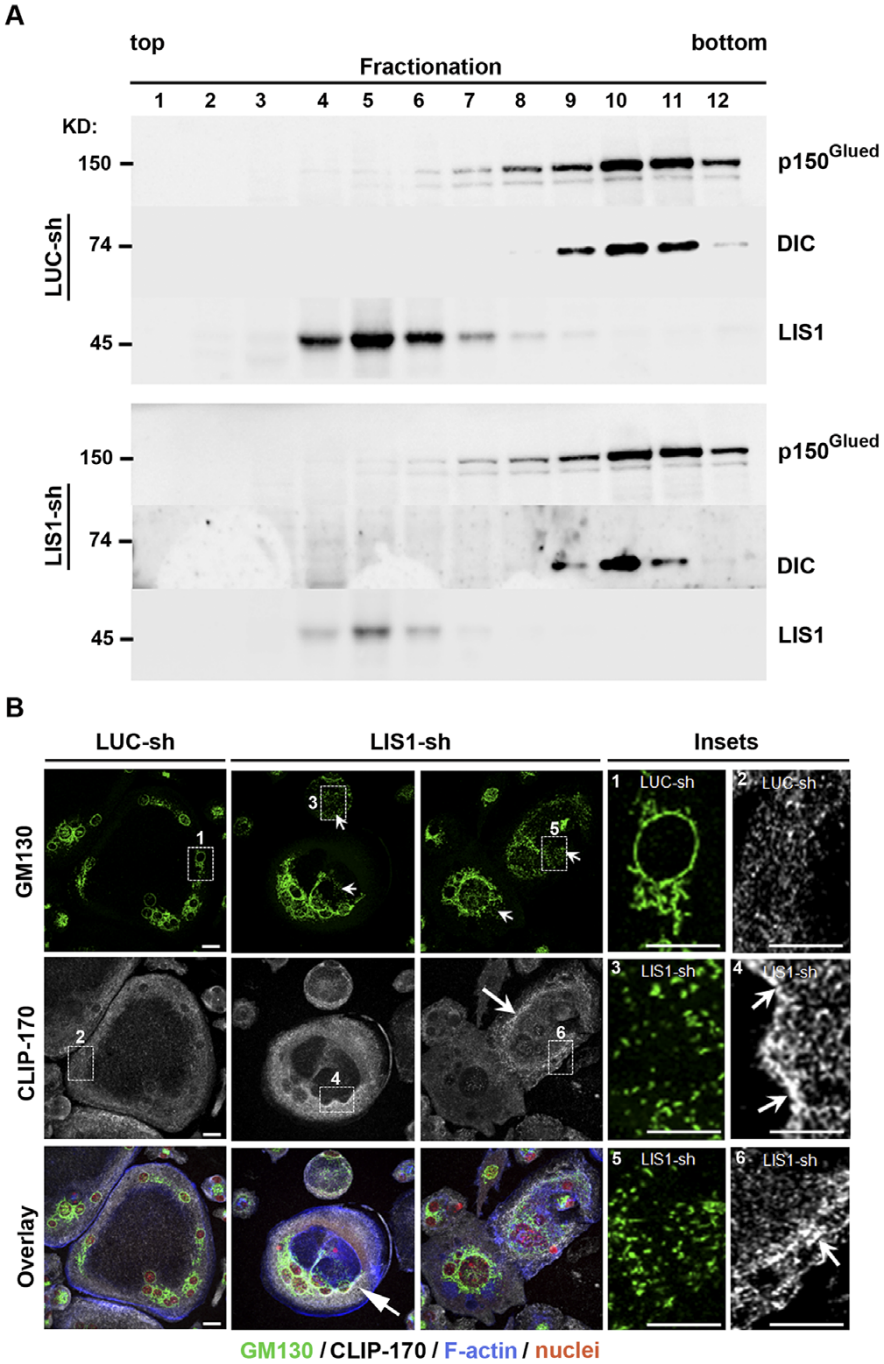
LIS1 modulates cytoplasmic dynein activity in osteoclasts. (**A**) Loss of LIS1 in osteoclasts does not affect dynein/dynactin complex integrity as measured by a sucrose gradient sedimentation assay. (B) Decreased LIS1 expression in osteoclasts causes dispersed Golgi apparatus (arrows in upper panels and inset 1, 3 and 5) and reduced transportation of CLIP170 to the periphery of osteoclasts. Arrows in the middle panels indicate an increased accumulation of CLIP170 around the nucleus.

Since organellar positioning and microtubule stability are intimately coupled to the dynein/dynactin motor complex we next examined the impact of LIS1 down-regulation on dynein/dynactin complex integrity as assessed by velocity density sedimentation assays. As shown in Figure 8A, no obvious differences were observed in the levels of DIC and p150^Glued^ in LIS1-depleted osteoclasts. Both proteins migrated to the same high-density fractions of the gradients in LIS1-depleted osteoclasts as compared to control cells, indicating that loss of LIS1 did not affect the integrity of dynein/dynactin complex, a finding consistent with recent studies [32]. The transportation of microtubule plus-end capping proteins such as EB1 and CLIP-170 to the cell periphery and the organization of the Golgi complex are mediated by LIS1-regulated dynein activity [36]–[38]. To further determine whether LIS1 regulates dynein function in osteoclasts we also examined the subcellular localization of GM130, a marker of Golgi complex, and EB1/CLIP170 in osteoclasts cultured on coverslips. As shown in Figure 8B, loss of LIS1 resulted in Golgi apparatus dispersion and fragmentation. While a fraction of EB1 and CLIP170 was observed to surround the nucleus, a significant amount of both proteins was found to localize at the periphery of well-spreading osteoclasts transduced with LUC-sh (Figure 7C and 8B left panels). Decreased LIS1 expression induced an increased level of EB1 and CLIP170 around the nucleus and there were a reduction of these two proteins at the cell periphery, suggesting that LIS1 plays an important role in regulating dynein function in osteoclasts (Figure 7C right panel and Figure 8B insets). Consistent with this notion, overexpression of p50 subunit (dynamintin) of dynactin complex, which has been shown to inhibit dynein/dynactin complex function, led to a similar change of EB1 distribution to that of LIS1-knockdown osteoclasts (Figure S6).

## Discussion

**Figure 9:**
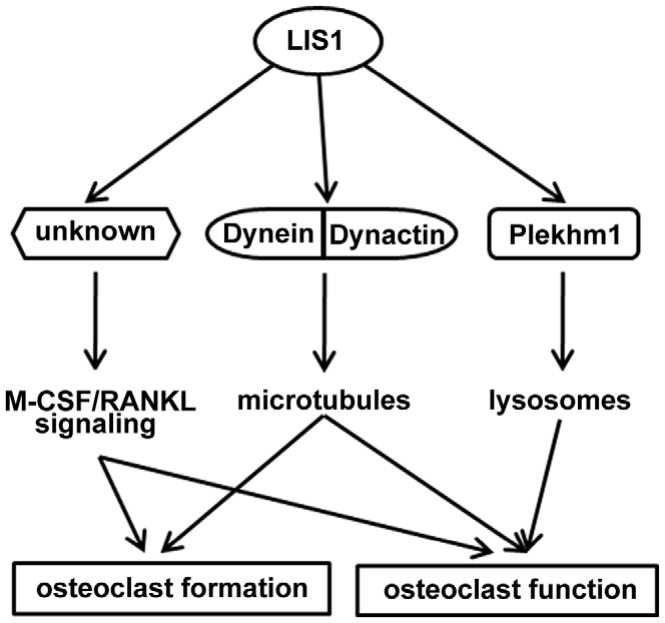
A schematic model for LIS1 in osteoclast formation and function.

LIS1 is crucial for neuronal migration, mitosis and intracellular transportation because of its role in regulating cytoplasmic dynein function [24], [25]. The evidence presented here demonstrates, for the first time, that LIS1 also plays an essential role in osteoclast formation and function via regulating several distinct but integrated pathways (Figure 9). First, as in neuronal cells, LIS1 regulates dynein/dynactin function and microtubule organization, which is critical for osteoclast activation and function. Second, LIS1 binds to Plekhm1, a newly identified osteoclast lysosome adaptor protein. Together with dynein/microtubule pathway, the interaction of LIS1 and Plekhm1 may be required for the special positioning of lysosomes and the delivery of Cathepsin K to the ruffled border membrane during osteoclastic bone resorption. Third, by a hitherto unknown mechanism(s), LIS1 modulates both the M-CSF and RANKL signaling pathways and hence regulates the proliferation, differentiation and survival of osteoclast precursors and mature cells.

Accumulating evidence indicates that microtubule organization/stabilization, controlled by microtubule acetylation, plays a critical role in cytoskeleton reorganization during osteoclast activation and function [10]–[13]. In this study, we have provided compelling data demonstrating that LIS1-regulated dynein function represents another important pathway essential for microtubule dynamics in osteoclasts. LIS1 interacted with dynein/dynactin complex, as revealed by its co-immunoprecipitation and co-localization with dynein and dynactin in mature polykaryons. Knock-down of LIS1 by shRNA led to a dramatic alteration of microtubule organization with a marked reduction in cortical microtubule penetration and a more focused microtubule network around the nucleus, without a significant change in the level of microtubule acetylation. Loss of LIS1 did not affect the integrity of dynein/dynactin complex since similar migration rate of dynein/dynactin complex was observed in a density-gradient of LIS1-depleted osteoclast lysates as compared to those of control cells. However, as in neuronal cells, LIS1 is essential for dynein/dynactin complex function in osteoclasts because loss of LIS1 resulted in dispersed Golgi apparatus, impaired motility of osteoclast precursors, and marked changes in the distribution of microtubule plus-end capping proteins, such as EB1 and CLIP170. Therefore, similar to the situation in neuronal and other cell types [32], [36], LIS1-regulated dynein function mediates the transportation of microtubule fragments and EB1/CLIP170 to the microtubule plus-ends at cell cortex which is required for the capture and stabilization of microtubules at the periphery of osteoclasts [39].

We have shown that LIS1 binds to the RUN and PH1 domain of Plekhm1 in osteoclasts. How LIS1-Plekhm1 interaction affects LIS1-mediated dynein function remains to be further characterized. We and others have shown that Plekhm1 is a lysosomal adaptor protein in osteoclasts and is functionally important for cathepsin K secretion and bone resorption [15] (Figure S2). Thus, it is likely that, by binding to LIS1, Plekhm1 may link lysosomes to microtubules in osteoclasts and mediates their transportation to the plus-ends of microtubules located at the cortex of the plasma membrane. LIS1 and its binding proteins, NDE1 and NDEL1, have been demonstrated to regulate dynein-dependent organelle distribution and positioning. However, the effect of LIS1 depletion on lysosome localization and transportation in osteoclasts is quite different from that in neuronal and other cells. While loss of LIS1 induces a dispersed pattern of lysosomes in other cells [32], [33], decreased level of LIS1 led to lysosome accumulation around the nucleus in osteoclasts. The underlying explanation of this distinct phenotype may lie in the differences between microtubule organizing center (MTOC) and localization of microtubule minus-ends in osteoclasts versus other cell types. In most cell types, there is a peri-nuclear located MTOC harboring the minus ends of microtubules. Loss of LIS1 leads to decreased dynein function which may reduce dynein-mediated microtubule minus-end oriented transportation of lysosomes [40]. Mammalian osteoclasts, however, do not have MTOCs during evolution [41]. The microtubule minus-ends are not nucleus-oriented and the direction of dynein-mediated transportation remains unclear.

Given that the major cellular function of LIS1 is attributed to its regulatory role in dynein and microtubules, thus, it is surprised that LIS1-depleted macrophages exhibited aberrant signaling pathways downstream of M-CSF and RANKL and decreased osteoclastogenesis. Although we cannot rule out the possibility that changes in cell physical properties such as cell shape and motility induced by abnormal microtubule organization in LIS1 depleted macrophages may affect cells response to M-CSF and RANKL, several other mechanisms might contribute to the modulation of LIS1 in M-CSF and RANKL signaling in osteoclasts. For example, earlier work has reported that LIS1 interacts with Syk [42], a non-receptor tyrosine kinase that is downstream of M-CSF, immunoreceptors with ITAM motif, calcium, and integrin signaling; and is critical for osteoclastogenesis in vivo and in vitro [43]–[46]. The decreased osteoclast formation in Syk^-/-^ macrophages can be rescued by high-dose M-CSF in vitro. Since high-dose of M-CSF and RANKL could not normalize osteoclast differentiation in LIS1-depleted cultures (Ye S and Zhao H unpublished observation) it is unlikely that Syk is a mediator of LIS1 in M-CSF and RANKL signaling. Both LIS1 and its binding protein NUDEL have been shown to modulate the activity of cdc42, a member of small GTPase Rho family, by direct interaction with cdc42 and its endogenous inactivator cdc42GAP, respectively [47], [48]. We have recently found that cdc42 is required for bone homeostasis by modulating M-CSF and RANKL signaling [49]. It is therefore possible that cdc42 plays a role in mediating LIS1's action on M-CSF and RANKL signaling. Another factor which may contribute to the regulation of LIS1 in M-CSF and RANKL signaling is PAF, an autocrine lipid messenger which is essential for osteoclast survival and activity [28]. Whether LIS1 interacts and regulates cdc42 and PAF in osteoclasts deserves further investigation in the future.

## Materials and Methods

### Antibodies and reagents

Commercially available antibodies were purchased from the following resources: rabbit polyclonal anti-Lissencephaly-1, mouse monoclonal anti-Dynein intermediate chain (74.1) and mouse monoclonal anti-Cathepsin K antibodies (Millipore); mouse monoclonal anti-HA antibody (Covance); mouse monoclonal anti-V5 antibody (Invitrogen); mouse monoclonal anti-GS28, anti-GM130, anti-p150^Glued^ and anti-EB1 antibodies (BD Transduction Laboratory); rabbit polyclonal anti-NFATc1 and anti-Dynein heavy chain antibodies (Santa Cruz); rabbit polyclonal anti-ERK1/2, mouse monoclonal anti-phospho-ERK1/2 (Thr202/Tyr204), mouse monoclonal anti-Akt (pan) (40D4), rabbit monoclonal anti-phosphor-Akt (Ser473)(193H12), rabbit polyclonal anti-JNK, mouse monoclonal anti-phospho-JNK (Thr183/Tyr185)(G9), rabbit polyclonal anti-IКB-α and mouse monoclonal anti-phospho-IКB-α (Ser32/36) (5A5) antibodies (Cell Signaling); mouse monoclonal anti-α-tubulin (DM1A) and anti-acetylated tubulin (611B1) antibodies (Sigma-Aldrich). Mouse anti-LIS1 monoclonal antibody was a generous gift from Dr. O Reiner (The Weizmann Institute of Science, Rehovot, Israel). CLIP170 antibody was kindly provided by Dr. N Galjart (Rotterdam, The Netherlands). The fluorescein-labeled secondary antibodies used in immunofluorescence were purchased from Jackson ImmunoResearch Laboratories. Alexa-488 conjugated phalloidin was obtained from Invitrogen. Hoechst 33342 was from Molecular Probes (Invitrogen). Hoechst 33258 was from Sigma-Aldrich. Peroxidase-conjugated wheat germ agglutinin was bought from Sigma-Aldrich. Protein A/G PLUS-agarose was from Santa Cruz. Cell culture media and L-Glutamine-penicillin-streptomycin solution were purchased from Invitrogen and Sigma-Aldrich, respectively. Fetal bovine serum was purchased from Hyclone.

### Bone marrow macrophage and osteoclast cultures

Bone marrow macrophages were prepared as described previously [50]. Briefly, whole bone marrow was extracted from tibiae and femora of 8–10 weeks old C57/BL6 mice (purchased from the Jackson Laboratory and were maintained according to guidelines of UAMS Institutional Animal Care and Use Committee, protocol number 3009) and incubated in red blood cell lysis buffer (150 mM NH_4_Cl, 10 mM KNCO_3_, 0.1 mM EDTA, and pH 7.4) for 5 minutes at room temperature. Bone marrow cells were collected by centrifugation and were plated in fresh α-10 medium (α-MEM, 10% heat-inactivated fetal bovine serum, 100 IU/ml penicillin, 100 µg/ml streptomycin and 2 mM glutamine) containing 1/10 volume of CMG 14–12 culture supernatant (which was equivalent to 130 ng/ml of recombinant M-CSF) in 10 cm petri-dish [51]. Cells were incubated at 37°C in 5% CO_2_, 95% air for 4-5 days. Fresh media and M-CSF were replaced every the other day. Pre-osteoclasts and osteoclasts were generated after three and five days culture of BMMs with 1/100 vol of CMG 14–12 culture supernatant and 100 ng/ml of recombinant RANKL, respectively.

### Cell proliferation and apoptosis Assays

BMMs were cultured with M-CSF for 3 days. Brdu was then added to the culture medium and incubated for 6 hours. BrdU incorporation rate was measured by the Cell Proliferation ELISA system (Amersham Biosciences) following the manufacturer's instructions. For the detection of osteoclast apoptosis, BMMs were culture with M-CSF and RANKL for 3 days. Pre-osteoclasts were either untreated or serum and cytokine starved for 3 hours. Caspase-3 activity was measured as previously reported [52]. Briefly, cells were lysed in 20 mM Tris-HCl (pH 7.5), 150 mM NaCl, 1 mM EDTA, 10 mM NaF, 1 mM sodium orthovanadate, 5 µg/mL leupeptin, 0.14 U/mL aprotinin, 1 mM phenylmethylsulfonylfluoride, and 1% Triton X-100. Protein concentration was measured using a Bio-Rad detergent–compatible kit (Bio-Rad). Lysates (100 µg protein) were incubated with 50 µM DEVD-AFC in 50 mM HEPES (pH 7.4), 100 mM NaCl, 0.1% CHAPS, 10 mM DTT, 1 mM EDTA, and 10% glycerol. The released fluorescent AFC was measured in a microplate fluorescence reader FL500 (Bio Tek Instruments) with excitation/emission wavelengths of 340/542 nm.

### TRAP and resorption pits stainings and medium CTx measurement

Bone marrow macrophages were cultured on 48-well tissue culture plate and bone slices in α-10 medium with 100 ng/ml RANKL and 1/100 volume of CMG14-12 supernatant for 4–5 days. The fresh medium and cytokines were changed at every the other day. The cells cultured on plastic dishes were fixed with 4% paraformaldehyde/PBS and TRAP was stained with a commercial kit (387-A, Sigma). Mature osteoclasts grown on bone slices were fixed with 4% paraformaldehyde/PBS for 20 minutes. After washing twice in PBS, the cells were removed from bone slices with a soft brush. The slices were then incubated with 20 µg/ml peroxidase-conjugated WGA-lectin (Sigma) for 30–60 min at room temperature. After washing in PBS twice, 0.52 mg/ml 3,3′-diaminobenzidine (Sigma) was added onto the slices for 30 minutes. Samples were mounted with 80% glycerol/PBS and were observed using a Carl Zeiss microscope equipped with a CCD camera [53]. The total resorbed area/bone slice was quantified using Osteomeasure software (Osteomeasure). Medium CTx-1 concentration was determined using CrossLaps for Culture ELISA kit (Immunodiagnosticsystems) following the manufacture's instruction.

### Lentivirus mediated shRNA expression

The RNAi sequence 5′-GCAACAGGATCTGAGACTA-3′ targeting mRNA of murine LIS1 was selected with RNAi designing online program from Dharmacon. The RNAi sequences 5′-CCATCTCTGAAGCAACAGGAT-3′ (LIS1a-sh) and 5′-GCAGATTATCTTCGTTCAAAT-3′ (LIS1b-sh) ligated in LKO.1 lentiviral vector were purchased from Sigma-Aldrich. A firefly *luciferase* shRNA was used as a control (5′- GCTTACGCTGAGTACTTCGA-3′). 293-T cells were co-transfected with LKO.1 gene transfer vector and virus packaging vectors, ΔH8.2 and VSVG using TransIT-LT1 transfection reagent (Mirus). Virus supernatants were collected 48 hours after transfection. BMMs were transduced with virus supernatant for 24 h in the presence of M-CSF and 20 µg/ml Protamine (Sigma-Aldrich). Cells were then selected in the presence of 100 ng/ml M-CSF and 6 µg/ml puromycin (Sigma-Aldrich) for 3 days [54].

### Retrovirus mediated BMMs transduction

The HA-tagged full-length of murine plekhm1 was amplified from an osteoclast cDNA library and the V5 tagged full length of murine LIS1 (PAFAH1b1) was amplified using pCMV-SPORT6-PAFAH1b1 (Thermal Open Biosystems) as a template by RT-PCR with Pfx DNA polymerase (Invitrogen). The cDNAs were cloned into the pMX-IRES-BSR retrovirus vector. The HA-tagged N-termimal fragment of plekhm1 (N), N-ter delta RUN domain, C-terminal fragment of plekhm1 (C) and C-ter delta PH1 domain were amplified by RT-PCR using pMX-BSR-mPlekhm1 as a template and cloned into the pMX-BSR retroviral vector. All the sequences were verified by DNA sequencing. The recombinant retroviral vectors were transiently transfected into Plat E packing cells using TransIT-LT1 transfection reagent (Mirus). Virus was collected 48 hours after transfection. BMMs were transduced with virus for 24 h in the presence of M-CSF and 20 µg/ml Protamine (Sigma-Aldrich). Cells were then selected in the presence of M-CSF and 2 µg/ml BSR (Calbiochem) for 3 days [30].

### Time-lapse video microscopy and cell motility assay

For the live cell imaging, 1.4 × 10^5^ of BMMs were plated onto Bioptechs chamber dishes and cultured with either 100 ng/ml M-CSF alone (macrophages) or 100 ng/ml M-CSF plus RANKL (pre-osteoclasts) for 3 days. The cells were imaged under phase contrast at 10X objective lens with the Bioptechs dish being heated to 37°C and 5% CO2/Air perfused into the chamber. Mineral oil was overlayed atop of the medium. Images were obtained using Scion imaging software at 6 images/hour. The 8-hour movements of ten motile osteoclast precursors from 48 images were tracked and analyzed with ImageJ MTrackJ plug-in (National Institutes of Health). Cumulative Length is depicted as total pixel number displaced over 48 images (8 hrs).

### GST pull-down assay

The cDNA fragments of individual domains of murine Plekhm1 were amplified by RT-PCR using pMX-BSR-mPlekhm1 as a template and were cloned in-frame into pGEX4T.1 vector. GST and GST fusion proteins were expressed in *Escherichia coli* BL21(DE3)plysS and purified on glutathione-Sepharose 4 Fast Flow beads (GE Healthcare). For GST pull-downs, 2 µg of GST and GST fusion proteins in 200 µl of immunoprecipitation (IP) buffer (20 mM Hepes pH 7.4, 120 mM NaCl, 5%glycerol, 0.5 mM EDTA, 1 mM NaVanidate, 5 mM NaF, 0.2% NP-40) were incubated with 20 µl of glutathione beads for 1 hour at 4°C. After washing twice with IP buffer, 2 mg of mature osteoclast lysate was added to the beads and incubated with rotation for 2 hours at 4°C. The beads were washed 5 times with IP buffer. The interacting proteins were eluted by boiling with 2× SDS gel loading buffer, and subjected to SDS-PAGE. Resolved proteins were detected by staining of gels with Coomassie blue and mass spectrometry or by immunoblotting with anti-LIS1 antibody [55].

### Immunoblotting and immunoprecipitation

Cultured cells were washed with ice-cold PBS and lysed in 1x RIPA buffer (Sigma) containing 1 mM DTT and Complete Mini EDTA-free protease inhibitor cocktail (Roche). After incubation on ice for 30 min, the cell lysates were clarified by centrifugation at 14,000 rpm for 15 min at 4°C. 10–30 micrograms of protein were subjected to 8 or 12% sodium SDS-PAGE gels and transferred electrophoretically onto PVDF membrane by a semi-dry system (Bio-Rad). The membrane was blocked in 5% fat-free milk/Tris-buffered saline-0.1% Tween 20 for 1 h and detected with primary antibodies at 4°C overnight followed by secondary antibodies conjugated with horseradish peroxidase (Santa Cruz Biotechnology, Santa Cruz, CA). After rinsing 3 times, the membrane was subjected to Western blot analysis with ECL detection reagent (Millipore).

For immunoprecipitation, osteoclasts were grown in 15-cm dishes with M-CSF and RANKL for 4–5 days as described above. Cells were washed in cold PBS and lysed on ice in H-buffer (20 mM HEPES, pH 7.4; 1% Triton X-100; 1 mM EDTA; 150 mM NaCl; 1 mM DTT and complete protease inhibitor cocktail from Roche). Lysates were incubated on ice for 30 min. The cell lysates were clarified by centrifugation at 14,000 rpm for 15 min. 1–2 mg of total protein were incubated with 2–5 µg primary antibodies for overnight with rotation at 4°C. Protein A/G agarose was then added and incubated with rotation for 3 h at 4°C. Immunoprecipitates were washed three times in lysis buffer, and the beads were boiled in 2x SDS sample buffer for 5 min. After centrifugation, proteins were separated by 8 or 10% SDS-PAGE gels.

### Immunofluorescence and confocal microscopy

Immunofluorescence was performed as previously described [56]. In brief, cells grown on glass slides in 24-well plate were fixed with 4% paraformaldehyde in PBS for 20 minutes, followed by permeabilization and blocking in PBS/0.2% BSA/0.1% saponin (PBSBS) for 30 minutes. The cells were then incubated with primary antibodies in 0.2% BSA/PBS for 2 hours. Primary antibody binding was visualized using fluorescent dye–conjugated secondary antibodies (Jackson ImmunoResearch Laboratories Inc.) in 0.2% BSA/PBS for 45 minutes. F-actin was stained with Alexa488-phalloidin. Samples were mounted with 80% glycerol/PBS. The nucleus was labeled with Hoechst 33258. Immunofluorescence-labeled cells were observed using a Carl Zeiss fluorescence microscope equipped with a CCD camera and analyzed using a Zeiss Spectral confocal laser scanning microscope equipped with Argon-Krypton lasers (LSM510 Zeiss Microsystems, Germany).

### Velocity Density Gradient Centrifugation of Dynein-Dynactin Complexes

Velocity density gradient centrifugation was performed as previously described in [57] with minor modifications. Briefly, ∼10^7^ osteoclastic cells were washed with ice-cold PBS, and scraped with 500 µl of lysis buffer (50 mM HEPES-KOH, pH 7.2, 150 mM NaCl, 1 mM MgCl_2_) supplemented with protease inhibitors: 1 mM phenylmethylsufonyl fluoride (PMSF), 1 µg /ml aprotinin, 1 µg /ml leupeptin and 0.7 µg /ml pepstatin (all from Sigma), and then homogenized by passing cells through a 25-gauge syringe (at least 10 strokes). Proteins were then cleared by centrifugation at 3000 rpm for 15 min at 4°C. 500 µl of the clarified supernatant was layered on top of 12 ml of 5–20% linear sucrose density gradient prepared in the lysis buffer. After centrifugation at 150,000 g for 18 h in a SW40 rotor (Beckman Coulter), 1 ml fractions were collected, acetone precipitated and equal amounts analyzed by SDS-PAGE and immunoblotting using mouse anti-p150^Glued^ (1∶1000), mouse anti-Dynein intermediate chain (IC74) (1∶1000) and rabbit anti-LIS1 antibodies.

### Statistics

Data of 2-group comparisons were analyzed using a 2-tailed Student's *t* test. For all graphs and in the text, data are represented as mean ± SD.

## Supporting Information

Figure S1 **Domain structure and constructs of mouse Plekhm1.**
(TIF)

Figure S2 **Plekhm1 is localized at lysosomes and the ruffled border membrane in osteoclasts and is essential for Cathepsin K secretion and bone resorption.** (A and B) Plekhm1 is associated with lysosomes in osteoclasts cultured on glass coverslips. (C) Plekhm1 is localized at the ruffled border membrane in resorbing-osteoclasts cultured on bovine bone slices. BMMs were transduced with a retroviral vector expressing the HA-tagged full length murine Plekhm1. Plekhm1, F-actin, and LAMP-2 (lysosome associated membrane protein 2) were labeled with a mouse monoclonal anti-HA antibody, phalloidin, and a rat monoclonal anti-LAMP-2 antibody, respectively. The cells were visualized by conventional (A and B) and confocal (C) fluorescent microscopes. (D) Plekhm1 mRNA and (E) protein expression were abolished by a lentivirus-mediated shRNA expression. (F) Plekhm1 knockdown osteoclasts form actin-rings normally as compared to control cells. (G) Cathepsin K secretion, (H) resorption pits formation and (I) medium CTx-I level were dramatically decreased in Plekhm1-depleted osteoclast cultures as compared to controls. Arrows in (G) showed the secreted Cathepsin K in the resorption lacunae circled by an actin-ring. Scale bars  = 10 µm. * in (I), p<0.05 vs LUC-sh by Student's t-test.
(TIF)

Figure S3 **An independent LIS1 shRNA inhibits LIS1 expression and osteoclast formation.** (A) Knockdown of LIS1 expression in macrophages by a second lentivirus-mediated shRNA. (B) LIS1 down-regulation attenuates multinucleated TRAP^+^ osteoclast formation. Scale bar  = 10 µm.
(TIF)

Figure S4 **LIS1 down-regulation increases pre-osteoclast apoptosis.** (A) LUC-sh and LIS1-sh transduced macrophages were cultured with M-CSF and RANKL for 2 days to generate pre-osteoclasts. The cells were then either un-treated or starved for 6 hours before fixation with 4% paraformaldehyde in PBS for 20 minutes. The nuclei were stained with Hoechst 33258. The debris of an apoptotic nucleus was shown by arrows. The scale bar  = 10 µm. (B) The numbers of apoptotic and total pre-osteoclasts in each group were counted under conventional fluorescent microscope. ** p<0.01 vs LUC-sh by Student's t-test.
(TIF)

Figure S5 **LIS1 and p150^Glued^ are localized at peri-nuclear cytoplasmic area and the peripheral podosome-belts.** (A) endogenous LIS1, (B) V5 tagged LIS1, and (C) endogenous p150^Glued^ in osteoclasts cultured on glass coverslips were stained with phalloidin and mouse monoclonal anti-LIS1, V5, and p150^Glued^ antibodies, respectively. The cells were visualized by conventional fluorescent microscope. Scale bar  = 10 µm.
(TIF)

Figure S6 **p50 dynamintin overexpression alters EB1 localization in osteoclasts.** Bone marrow macrophages were transduced with either empty vector (pMX) or retroviral vector expressing p50 dynamintin (pMX-p50) and cultured with M-CSF and RANKL for 5 days to generate mature osteoclasts on glass coverslips. The cells were then fixed and labeled with anti-EB1 monoclonal antibody. Scale bar  = 10 µm.
(TIF)
