# Supplementary figures and images for: LIS1 Regulates Osteoclast Formation and Function through Its Interactions with Dynein/Dynactin and Plekhm1
Source: PLoS One. 2011 Nov 3;6(11):e27285. doi: 10.1371/journal.pone.0027285 (PMC3207863; doi:10.1371/journal.pone.0027285)

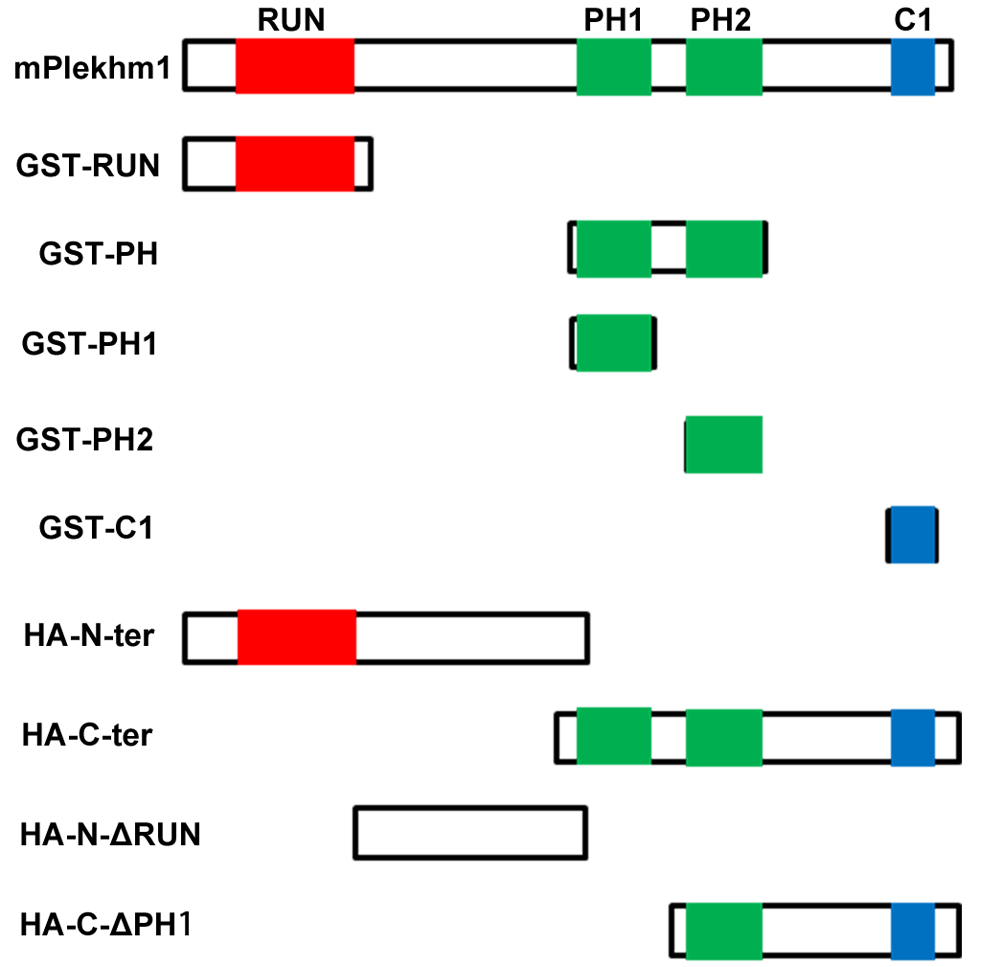

Supplement: Figure S1 — Domain structure and constructs of mouse Plekhm1. (TIF) [file pone.0027285.s001.tif]

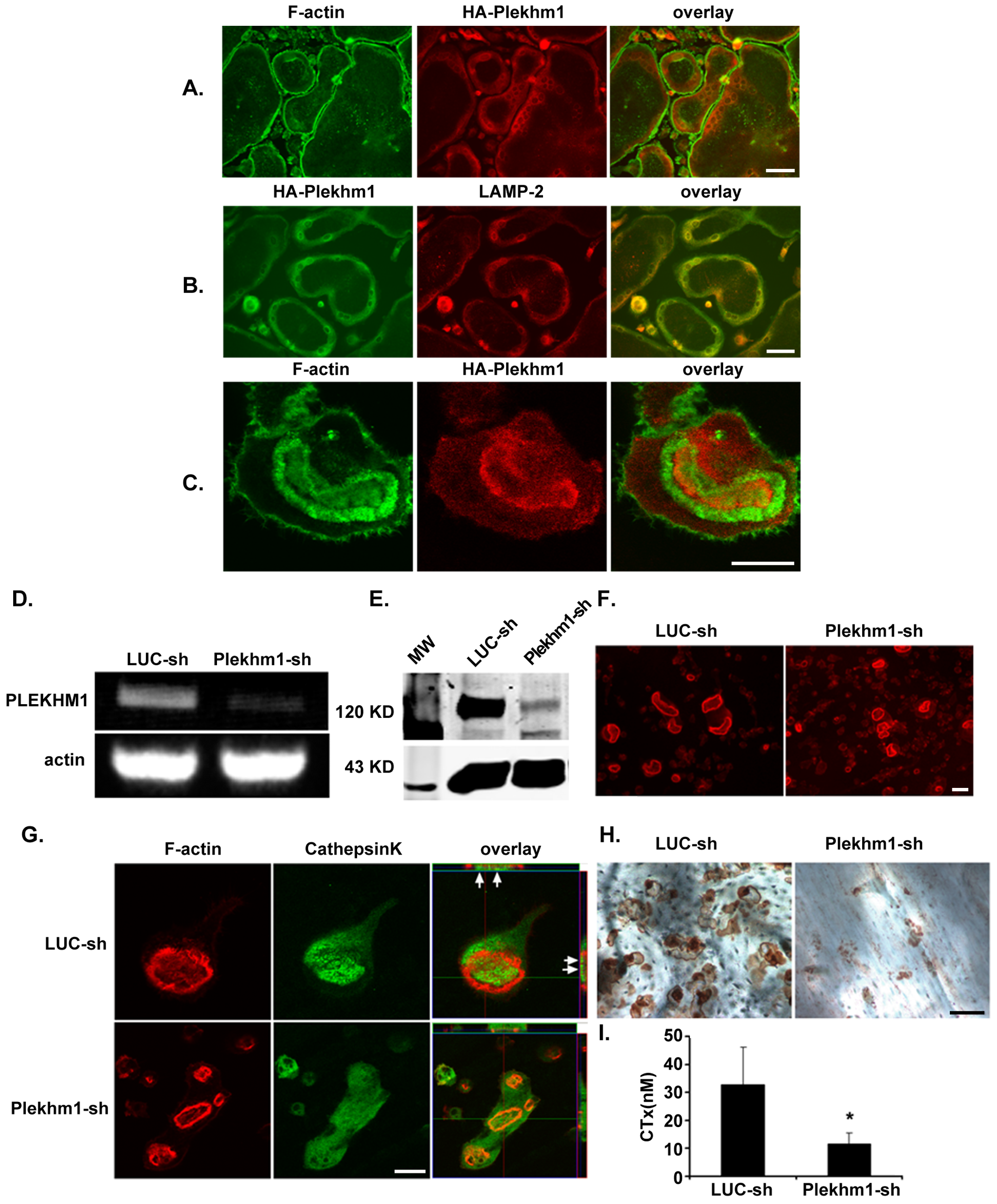

Supplement: Figure S2 — Plekhm1 is localized at lysosomes and the ruffled border membrane in osteoclasts and is essential for Cathepsin K secretion and bone resorption. (A and B) Plekhm1 is associated with lysosomes in osteoclasts cultured on glass coverslips. (C) Plekhm1 is localized at the ruffled border membrane in resorbing-osteoclasts cultured on bovine bone slices. BMMs were transduced with a retroviral vector expressing the HA-tagged full length murine Plekhm1. Plekhm1, F-actin, and LAMP-2 (lysosome associated membrane protein 2) were labeled with a mouse monoclonal anti-HA antibody, phalloidin, and a rat monoclonal anti-LAMP-2 antibody, respectively. The cells were visualized by conventional (A and B) and confocal (C) fluorescent microscopes. (D) Plekhm1 mRNA and (E) protein expression were abolished by a lentivirus-mediated shRNA expression. (F) Plekhm1 knockdown osteoclasts form actin-rings normally as compared to control cells. (G) Cathepsin K secretion, (H) resorption pits formation and (I) medium CTx-I level were dramatically decreased in Plekhm1-depleted osteoclast cultures as compared to controls. Arrows in (G) showed the secreted Cathepsin K in the resorption lacunae circled by an actin-ring. Scale bars = 10 µm. * in (I), p<0.05 vs LUC-sh by Student's t-test. (TIF) [file pone.0027285.s002.tif]

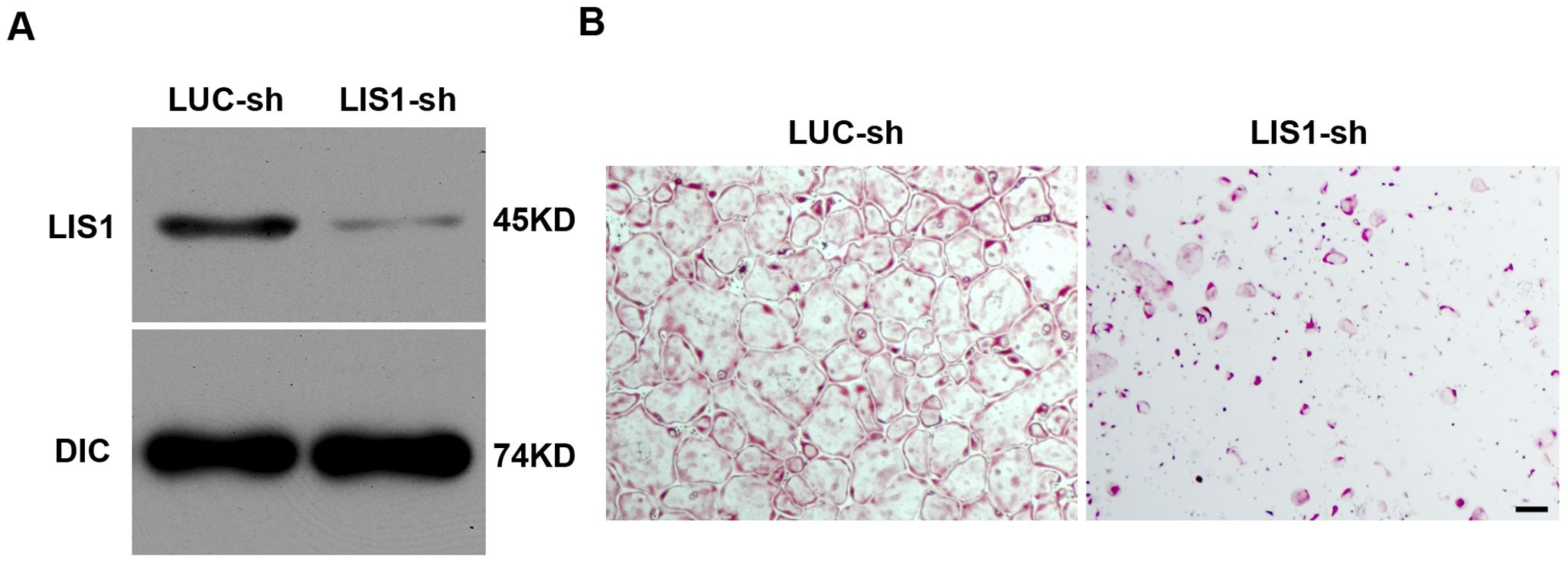

Supplement: Figure S3 — An independent LIS1 shRNA inhibits LIS1 expression and osteoclast formation. (A) Knockdown of LIS1 expression in macrophages by a second lentivirus-mediated shRNA. (B) LIS1 down-regulation attenuates multinucleated TRAP+ osteoclast formation. Scale bar = 10 µm. (TIF) [file pone.0027285.s003.tif]

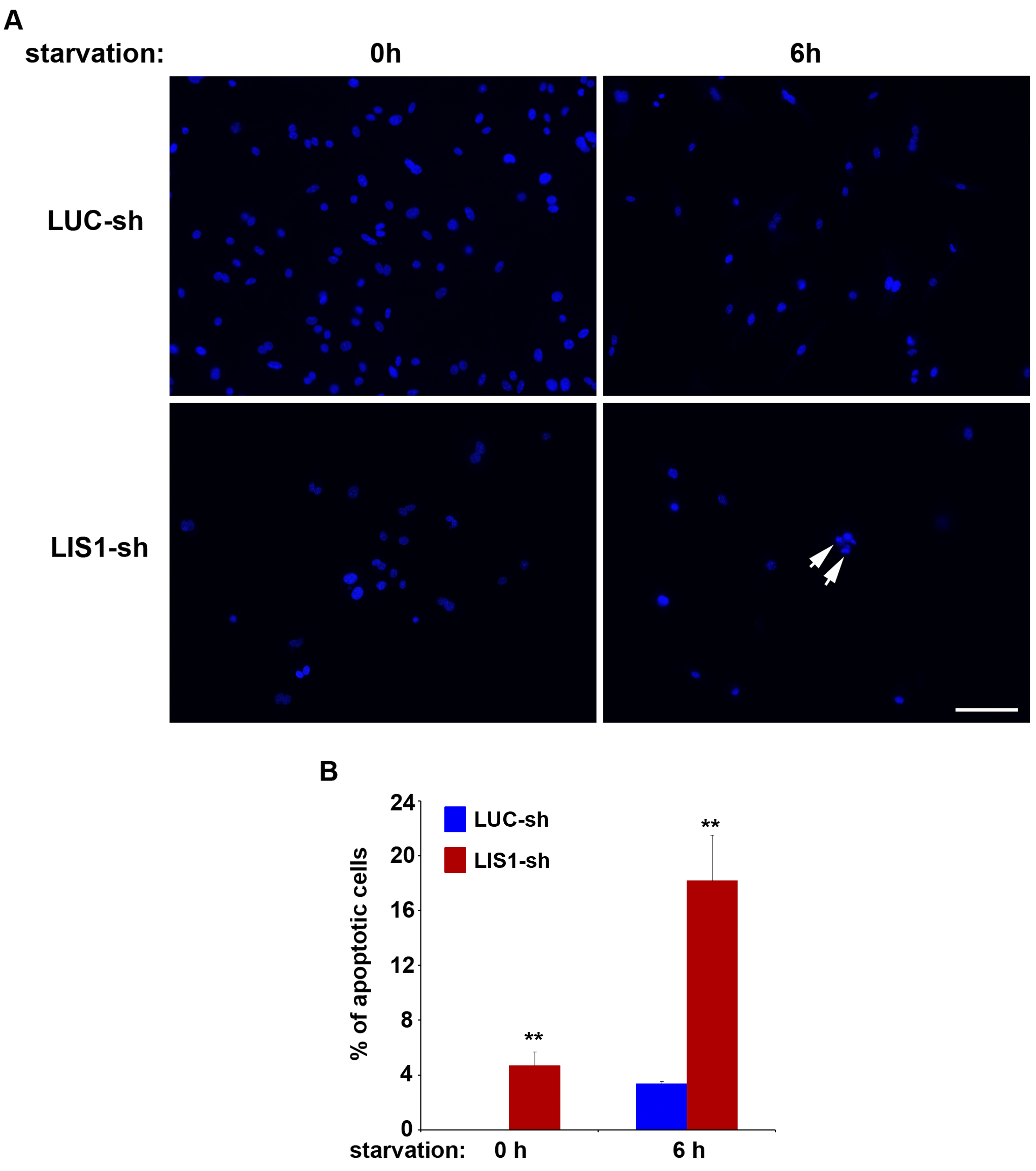

Supplement: Figure S4 — LIS1 down-regulation increases pre-osteoclast apoptosis. (A) LUC-sh and LIS1-sh transduced macrophages were cultured with M-CSF and RANKL for 2 days to generate pre-osteoclasts. The cells were then either un-treated or starved for 6 hours before fixation with 4% paraformaldehyde in PBS for 20 minutes. The nuclei were stained with Hoechst 33258. The debris of an apoptotic nucleus was shown by arrows. The scale bar = 10 µm. (B) The numbers of apoptotic and total pre-osteoclasts in each group were counted under conventional fluorescent microscope. ** p<0.01 vs LUC-sh by Student's t-test. (TIF) [file pone.0027285.s004.tif]

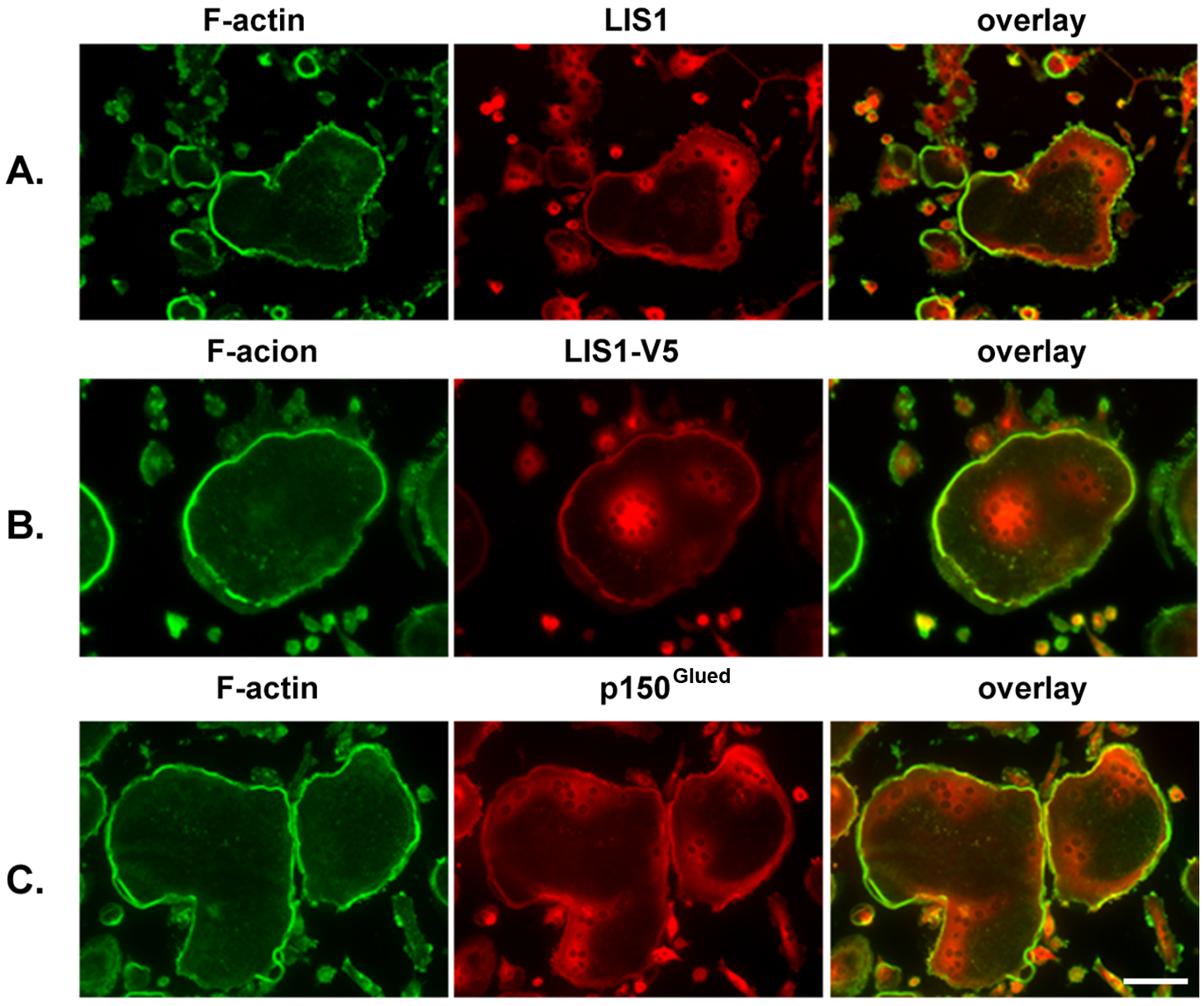

Supplement: Figure S5 — LIS1 and p150Glued are localized at peri-nuclear cytoplasmic area and the peripheral podosome-belts. (A) endogenous LIS1, (B) V5 tagged LIS1, and (C) endogenous p150Glued in osteoclasts cultured on glass coverslips were stained with phalloidin and mouse monoclonal anti-LIS1, V5, and p150Glued antibodies, respectively. The cells were visualized by conventional fluorescent microscope. Scale bar = 10 µm. (TIF) [file pone.0027285.s005.tif]

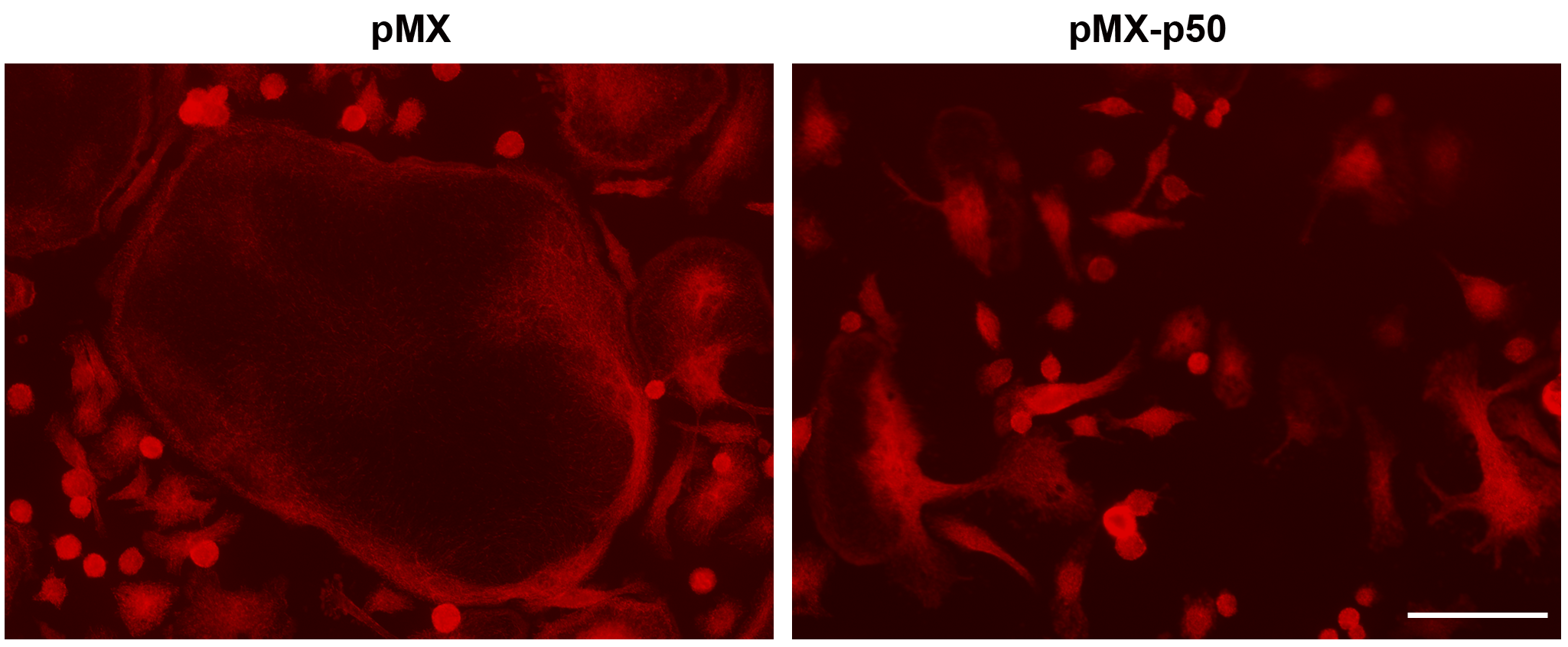

Supplement: Figure S6 — p50 dynamintin overexpression alters EB1 localization in osteoclasts. Bone marrow macrophages were transduced with either empty vector (pMX) or retroviral vector expressing p50 dynamintin (pMX-p50) and cultured with M-CSF and RANKL for 5 days to generate mature osteoclasts on glass coverslips. The cells were then fixed and labeled with anti-EB1 monoclonal antibody. Scale bar = 10 µm. (TIF) [file pone.0027285.s006.tif]
